# P-414. From Anecdotal to Analytical: Correlating Self-Reported *Norovirus*-Like Illness with Epidemiological Data

**DOI:** 10.1093/ofid/ofae631.615

**Published:** 2025-01-29

**Authors:** Patrick Quade, Lee-Ann Jaykus, Benjamin Chapman, Jeremy Faircloth, Rebecca Goulter

**Affiliations:** Iwaspoisoned.com; North Carolina State University, Raleigh, North Carolina; North Carolina State University, Raleigh, North Carolina; North Carolina State University, Raleigh, North Carolina; North Carolina State University, Raleigh, North Carolina

## Abstract

**Background:**

Human norovirus is a leading cause of foodborne illness globally, with hallmark symptoms of vomiting and diarrhea. The Norovirus Sentinel Testing and Tracking (NoroSTAT) network is a collaborative network of select U.S. state public health departments and the U.S. CDC, designed to collect epidemiologic and laboratory data on suspected or confirmed norovirus outbreaks. The global crowdsourcing website Iwaspoisoned.com (IWP), established in 2009, provides a means by which the public can self-report foodborne disease-like symptoms in real-time, world-wide. The value of crowdsourced data in relation, or complementary, to classic epidemiologic approaches, is largely unknown.

This study aims to investigate correlations between self-reported norovirus-like illness from IWP and norovirus epidemiological data from NoroSTAT.

**Methods:**

The analysis was limited to reports between August, 2018 and May, 2022. NoroSTAT data were obtained from their website and internet archives. Data was extracted directly from the IWP database, with the following inclusion criteria: (i) symptoms of both vomiting and diarrhea reported together (used as a proxy for norovirus-like illness); and (ii) reports originating from a NoroSTAT-participating state. Reports were excluded if spam or duplicate. Associations were investigated using Pearson Correlation and Granger Causality tests using the Minitab (State College, PA) software package.

**Results:**

The Pearson Correlation value of 0.669 was highly significant (p< 0.001), indicating a strong linear relationship between IWP and NoroSTAT dataset outcomes (those being time and number of reports/cases). The Granger Causality analysis indicated a highly significant directional correlation between the two datasets (p=0.003).

**Conclusion:**

There was a strong positive correlation between self-reported norovirus-like illness trends reported to the IWP crowdsourcing website, and NoroSTAT-reported outbreaks over the same timeframe. The alignment of the crowdsourced symptom data with public health surveillance lends credibility to the value of curated self-reporting as a complementary signal for detecting and perhaps predicting emerging norovirus epidemiological reports over time.

**Disclosures:**

**All Authors**: No reported disclosures

